# Telangiectatic osteosarcoma of the thoracic vertebra: a case report and literature review

**DOI:** 10.3389/fonc.2025.1537611

**Published:** 2025-04-17

**Authors:** Chen Wang, Xirong Fan, Kai Zhang, Wen Xue, Guoquan Li, Qilong Wang, Fadong Gou, Yi Zhou, Wenbo Xing, Chenjian Wang

**Affiliations:** ^1^ The 1st Clinical Medicine College, Gansu University of Chinese Medicine, Gansu, Lanzhou, China; ^2^ Gansu Provincial Hospital, Gansu, Lanzhou, China; ^3^ Qingshui County People’s Hospital, Gansu, Tianshui, China

**Keywords:** telangiectatic osteosarcoma, thoracic vertebrae, case report, literature review, bone tumors

## Abstract

Telangiectatic osteosarcoma (TOS) is a rare high-grade malignant tumour found primarily in the metaphysis of long bones, but can also occur in the pelvis, spine and skull. Characterized by extensive vascular dilatation and aggressive behavior, TOS often has a poor prognosis and may be misdiagnosed due to similarities with other lesions. This report presents a case of TOS of the thoracic vertebrae and reviews the relevant literature to enhance clinical awareness. A 44-year-old woman presented with chest and back pain of worsening over two months, accompanied by lower limb numbness and incomplete paralysis. Initial treatment for “low back pain” proved ineffective. Imaging showed osteolytic destruction at T10, which led to surgery, and the tumour was classified as Tomita type III. The tumour was surgically resected, showing vascular proliferation and malignant stromal cells, confirming the pathological diagnosis of TOS. However, she developed complications requiring further surgery for recurrent problems, where a hematoma was discovered. Despite interventions, the patient’s condition deteriorated, leading to the diagnosis of pulmonary metastasis and eventual death three months after the second surgery. This case highlights the need for increased awareness and accurate diagnosis of TOS, particularly in atypical presentations.

## Introduction

Telangiectatic osteosarcoma (TOS) is a rare and highly aggressive form of osteosarcoma. Epidemiological data showed that TOS mainly occurred in adolescents, with a median onset age of 17.5 years, and showed a significant gender preference, with a male and female incidence ratio of about 2:1 (1). The disease has a typical anatomical distribution, with about 42% of cases involving the distal metaphyseal of the femur and a small number of cases found in the pelvis, sternum, spine, or skull (1).Originating in mesenchymal cells within the bone marrow, the tumour is highly aggressive and metastatic, and the prognosis is often poor. Pathologically, TOS is characterized by a “blood bag like” (1)cystic structure, with microscopically visible blood-containing lacunae separated by fibrous septa, surrounded by a highly heterotypic interstitial cell population, and lacking the sclerotic neoplastic bone formation seen in traditional osteosarcoma. This unique pathological feature makes it necessary to rely on a comprehensive analysis of multimodal techniques in the imaging diagnosis of TOS. On X-ray images, TOS often presents as osteolytic destruction with cortical dilatation (2). A CT scan can identify the characteristic liquid-liquid plane and incomplete crust cortex (3), while an MRI can clearly show the extent of soft tissue infiltration and irregular cystic structure (4).At present, focal resection/amputation is the most commonly used in the clinical practice of TOS, and adjuvant chemotherapy also plays a vital role in the treatment. Spinal telangiectatic osteosarcoma (TOS) is rare, accounting for only 0.08% of all primary osteosarcoma (5, 6). It accounts for 2% of all primary vertebral osteosarcoma cases. Only seven reports were retrieved, and the survival time of patients ranged from 4 to 48 months. Currently, the management of spinal TOS faces a triple challenge. First, the complexity of the anatomy limits the recognition of image features - the bony structure of the vertebral body may mask the typical “blood bag” cystic degeneration and the liquid-liquid plane of the TOS. Second, tissue biopsies can be false-negative: the lesion area is often masked by reactive callus or old bleeding, making malignant stromal cells challenging to identify under the microscope. Third, current treatment options lack sufficient evidence-based support: Clinical studies have shown that long bone TOS is highly sensitive to adjuvant chemotherapy (5-year survival rate of 68%). At the same time, spinal TOS may lead to decreased penetration of chemotherapy agents due to dural invasion and heterogeneity of blood supply. The mean survival of spinal TOS was 16.4 months.

In this paper, we report a case of thoracic vertebra telangiectatic osteosarcoma (TOS) and provide a detailed review of the literature on this tumor, with the aim of enhancing clinical understanding and awareness.

## Case description

First admission: The patient is a female, aged 44. The primary causes of admission were “persistent dull pain in the chest and back for 2 months, increased numbness and weakness in both lower limbs, and incomplete paralysis for 1 week.” The patient was diagnosed with “low back pain” at a nearby hospital two months prior to admission after experiencing dull, continuous discomfort in the chest and back that had no apparent cause. The patient received cupping and gua sha, along with oral anti-inflammatory analgesics, but the symptoms persisted. A week before admission, the patient had worsening back and chest discomfort, particularly at night, as well as lower limb numbness and weakness, partial paralysis, and urine incontinence. The patient was admitted to our hospital to investigate the cause of her incomplete paralysis after the local hospital performed catheterization and a spinal X-ray examination that revealed no visible abnormalities.

Physical examination admission: Temperature: 36.3°C P = 85 x min R = 18 x min Blood pressure: 128/80 mmHg. The patient experienced a painful face, local tenderness and knock pain with the T10 spinous process in the center, numbness in both lower limbs, significant muscular strength grade III, hypotonia, and a sensory disturbance plane at the umbilical level when they arrived at the hospital in a flat automobile. Ankle clonus was negative, and the knee and Achilles tendon hyperreflexes were physiological responses. Bilateral pathological reflexes include the negative Babinski, Gordon, Chaddock, and Oppenheim signs. Both upper limbs’ sensations, movements, and reflexes were normal. Frankel graded it a D, and the VAS score was 9.

Imaging analysis: X-ray films revealed no discernible aberrant alterations, however each vertebral body had modest bone hyperplasia (**Supplementary Figures S1A, B**).CT scan showed low-density sacs and compartments along with core osteolytic degeneration of the T10 vertebrae (**Supplementary Figures S3A, B**).On MRI, the thoracic 10 vertebrae were found to be somewhat flattened, with lengthy T1 and T2 signals, high-fat pressure signals, and lesions that protruded backward and squeezed the dural sac (**Supplementary Figures S2A–E**).Bone scan revealed no aberrant concentration or defect in the remaining bone tissue but abnormal radioactivity in the T10 vertebrae. Initial diagnosis: Tomita type III primary spinal tumor with partial paralysis.

The T10 tumor was effectively removed under general anesthesia, complete vertebral resection, spinal canal decompression, intervertebral titanium mesh bone fusion, and internal fixation using a pedicle nail rod system after departmental debate.1500 ml of blood was lost during surgery, 800 ml of red blood cells, and 400 ml of plasma were injected. Fish-like alterations, soft, dark gray tissue, and a distinct border between the surrounding tissues were noted intraoperatively in the spinal canal, 3.5 cm beyond the dural level of the T9-10 vertebrae. Cortices were intact when the adnexa and T10 vertebrae were removed. The shell-like vertebrae were packed with tissue that resembled clots and fractured bones. The incision healed in one stage.

The postoperative pathological description of the tumor tissue included blood vessels, vascular lumen of different sizes, some dilated in irregular sacs, residual irregular bone trabeculae between the tube walls, scattered deposits containing hemosiderin, diffuse proliferation of numerous myofibroblasts, osteoclastic multinucleate giant cells and malignant stromal cells, local callus formation, and varying amounts of osteoid tissue (**Supplementary Figure S4A**). Immunological analysis: CD34 (microvascular endothelium +, densely organized), P53 (partial +), and Ki-67 (index 15%–20%).The pathological diagnosis is TOS. The patients were given large dosages of methotrexate, doxorubicin, cisplatin, and cyclophosphamide as part of chemotherapy for six to ten months. The patient was braced following surgery, and two weeks later, the primary muscle strength of the lower limbs was restored to grade IV. The patient began exercising independently on the ground after achieving a VAS score of three.

Secondary admission: Three months after surgery, the patient had defecation problems, sensory and motor impairments in both lower limbs, and a readmission to the hospital. Physical evaluation: 36.8°C is the temperature. R = 22 x min, and P = 80 x min Blood pressure:112/78mmHg;During the professional physical examination, a 15-cm longitudinal healing surgical scar was observed in the center of the back. Percussion aches and local tenderness were felt in the spinous process of the T10 vertebra. Both lower limbs showed zero primary muscle strength, increased muscular tension, and a discernible loss of cutaneous sensibility and numbness below the umbilical level. The joints in the lower limbs could only move passively instead of actively. The Babinski sign, patellar clonus, and ankle clonus were all positive, and the knee and ankle reflexes were hyperactive. Frankel’s rating is an A. Recurrence of TOS without total paralysis was the initial diagnosis. Pertinent assessments were completed. Thoracic canal decompression and spinal exploration were performed under emergency general anesthesia. Throughout the therapy, the dural membrane of the T10 spinal segment was densely covered in soft tissue, including hematoma, which was prone to bleeding when handled. After the wound was properly removed, it was closed using negative pressure drainage. Four hundred milliliters of red blood cells and 400 milliliters of plasma were transfused following the 1000 milliliters of blood lost during surgery. Frankel grade: The incision healed in a single step. Postoperative pathological description: unplastic tissue (epidural hematoma), gray, brown, and red. Its microscopic features include diffuse polygonal cells with irregular nuclei, a lot of chromatin and granules, some nucleoli, easy-to-see mitosis, a lot of cytoplasm and powder staining, frequently multi-nucleated giant cells, a lot of small interstitial blood vessels, congestion and bleeding, and coagulation necrosis in some areas (**Supplementary Figure S4B**). Among the pathological diagnoses are telangiectatic osteosarcoma, CD31(-), Ki-67 (index: 40%–50%), and FacterVIII (Microvascular endothelium +). Lung metastases led to the patient’s mortality at the second postoperative follow-up; postoperative paraplegia did not improve, and the patient ceased receiving therapy three months after surgery.

## Discussion

In 1903, Gaylord first proposed the theory of telangiectatic osteosarcoma (TOS). Matsuno (7) named telangiectatic osteosarcoma and established diagnostic criteria for it in 1976.It was verified as TOS in 2002 by the WHO’s classification of bone tumours (8). With a male-to-female ratio of 2:1 and an average onset age of 17.5 years (with a range of 15 to 20 years), this uncommon and severely malignant osteosarcoma affects children, teenagers, and young adults. The most prevalent anatomical site is the distal metaphysis of the femur (42%), which is followed by the proximal tibia (17%), proximal humerus (9%), and proximal femur (8%) (1). Given the short duration and rapid progression of the condition, it is important to be mindful of the potential for misinterpretation in clinical settings. Telangiectatic osteosarcoma’s exact etiology and risk factors are unknown.

Only 0.08% of all primary osteosarcomas are spinal telangiectatic osteosarcomas (TOS) (5, 6). It makes up 2% of all instances of primary vertebral osteosarcoma. Seven cases of spinal TOS were found, six of which were male and one of which was female, according to the Pubmed database. The patients’ ages ranged from 8 to 57 years old, with an average of 27.0 years. There were 6 cases of thoracic vertebra (1 case with lumbar vertebra) and 1 case of cervical vertebra. Between one and forty-eight months passed between the beginning and the diagnosis (9–12). Clinical presentation: The most common symptoms of telangiectasia osteosarcoma are local discomfort and/or soft tissue mass; the clinical presentation of telangiectasia and conventional osteosarcoma is identical.

Imaging findings: On X-ray films, TOS was mainly recognized by lytic and dilatant bone injury with an oval or irregularly contoured destruction zone (2). The CT scan revealed low density, unstrengthened areas, flat areas of cystic fluid fluid (3), and uneven tissue density in the area where the tumour had been destroyed. The bone shell was frequently missing, and the bone cortex displayed dilatant and crusty alterations. There was periosteum hyperplasia, a sizable lesion area, and an ambiguous lesion boundary. The degree and boundaries of soft tissue mass and bone loss are easily discernible on MR. The obvious and unexpected nature of cystic alterations substantially facilitates diagnosis (4).

Pathological findings: The bulk of the neoplasm, as it is visible to the naked eye, is a cystic formation in the medullary cavity that is partially filled with clots and resembles a “bag of blood” (1) with no discernible tumour tissue or hardened bone growth. Thin spacer phases that reveal empty or blood-filled sacs in the tumour tissue divide big tumours with smaller sacs under a microscope. A cavernous vascular area composed of fibrous septa may be seen under a microscope, and big cells that resemble osteoclasts and malignant stromal cells encircle the capsular cavity (2, 4).

TOS diagnostic standards (5) (13): Diagnostic criteria according to the WHO classification of soft tissue and bone tumours (5th edition) 1. imaging features of a bone tumour 2. osteoid matrix with neoplastic bone formation 3. permeative and destructive growth pattern. In addition, the following criteria are required: empty or blood-filled cystic spaces separated by septa. As with conventional osteosarcoma, the following histological criteria are desirable: 1. high-grade atypia of tumour cells,2. frequent atypical mitotic figures.

Diagnostic differentiation for TOS (14): ① Aneurysmal bone cyst: Aneurysmal bone cysts typically exhibit dilatant changes on X-ray images, with no soft tissue mass, periosteal reaction, or other malignant signs. Malignant indicators of TOS include soft tissue mass, periosteal response, and bone degeneration. Histologic TOS revealed the presence of clear osteosarcoma in the blood space and solid area, accompanied by fibroblastoid hyperplasia of the capsule wall. However, aneurysmal bone cysts were not observed. ② Giant cell tumour of bone: the tumour has large megakaryocytes, multiple nuclei, and a uniform distribution of multinucleated giant cells; it does not have small multinucleated giant cells distributed along the sac wall in TOS, nor does it have a blood conelike structure. Malignant tumours typically do not exhibit these characteristics, such as erosion of cortical bone and unclear lesion boundaries. Furthermore, research (3, 14) highlights the need to distinguish it from other conditions, such as hemangioma, eosinophilic granuloma, plasmacytoma, and osteolytic osteosarcoma.

Telangiectatic osteosarcoma is thought to have a poor prognosis and clinical result. Focused resection/amputation is currently the most common treatment for TOS in clinical practice, while adjuvant chemotherapy is also essential. Research has revealed that Huvos (15) first observed that TOS was particularly sensitive to chemotherapy. They hypothesized that the increased vascularity of the tumour increases drug delivery to the tumour site; therefore, TOS may be more chemoresponsive than other osteosarcoma subtypes. The five-year survival rate for long-bone telangiectatic osteosarcoma is 68% (16, 17) when adjuvant treatment is used. Five patients received adjuvant chemotherapy among the seven cases of post-resection TOS that have been recovered thus far. Cisplatin, carboplatin, methotrexate, doxorubicin, and ifosfamide are examples of chemotherapy medications that are often utilized. With an average survival duration of 16.4 months, ranging from 4 to 48 months, these cases had a much worse survival rate than individuals with long bone TOS (18). When used alone, radiotherapy has a limited effect and is not effective against osteosarcoma. There have been no reports of radiotherapy in TOS.

In this instance, a 44-year-old woman with spinal TOS was first diagnosed with incomplete paralysis and a primary spinal tumor. With T10 complete spinal resection, Tomita classification type III was carried out, and T10 TOS was the postoperative diagnosis. After receiving high-dose chemotherapy consisting of methotrexate, doxorubicin, cisplatin, and cyclophosphamide, the patient’s lower limb muscle strength returned to grade IV two weeks after surgery, and they began exercising independently. She died from lung metastases six months after surgery. Following total spinal excision, normal neurological function was restored. Full decompression was a crucial component of the treatment since the spinal cord damage caused by TOS was mostly caused by long-term chronic compression. The patient’s lifetime was less than the average for spinal TOS patients reported in the literature because of the absence of preoperative adjuvant chemotherapy, irregular postoperative chemotherapy, and treatment termination after recurrence and lung metastases.

Based on the above, and in combination with only eight cases of spinal telangiectatic osteosarcoma (TOS) reported worldwide (including this case), we propose three seminal hypotheses:

1. Elderly patients may have unique molecular characteristics: the postoperative pathological results of this case showed P53 (partial +), suggesting a tendency to Li-Fraumeni syndrome. In addition, this patient was significantly older than the average age of onset of TOS, which could lead to an increased risk of subsequent recurrence of the disease.
2. Rapid recurrence after total vertebrae resection may be related to intraoperative mechanical dissemination: ①The unique “empty shell sign” of TOS (showing cortical integrity on CT) may lead surgeons to underestimate the aggressiveness of the tumour. Although the tumour and its surrounding tissues were resected entirely in this case (pseudo envelope intact), postoperative pathology revealed microvascular penetration (CD34 microvascular endothelium +, densely packed) in the fibrous septa, suggesting the presence of subclinical satellite foci. This indicates potential signs of tumour metastasis, but no further management is taken, and it may be related to postoperative lung metastasis. ② The operation in En-bloc may induce microtumor thrombolysis. The recurrence of the lesion in this case was a ring-wrapped dural sac and showed non-focal growth, suggesting that the tumor might spread along the cerebrospinal fluid space, a feature that may be related to lung metastasis in this case.
3. Traditional chemotherapy may accelerate the vascular mimicry process of spinal TOS: Ki-67 (index:40%-50%) was found in the secondary pathology, which was more than double that in the first pathology, indicating the expansion of chemotherapy-resistant clones. In addition, P53 suggests acquired TP53 mutations, which may be associated with chemotherapy resistance. These results indicate that conventional chemotherapy regimens for osteosarcoma may not be appropriate for this patient, and replacement of chemotherapy regimens should be considered. However, in this case, the patient chose to abandon follow-up treatment after the second operation, which may be closely related to postoperative recurrence and lung metastasis.

## Conclusion

Telangiectatic osteosarcoma is an aggressive and rare variant of osteosarcoma. The occurrence of TOS in the spine is rare, and definitive diagnosis needs to be combined with imaging features and histopathology. The treatment of surgical excision and adjuvant chemotherapy has not improved. Clinicians should enhance their understanding of the disease to avoid missed diagnoses or misdiagnoses.

## Data Availability

The datasets presented in this study can be found in online repositories. The names of the repository/repositories and accession number(s) can be found in the article/**Supplementary Material**.
