# Supplementary figures and images for: Telangiectatic osteosarcoma of the thoracic vertebra: a case report and literature review
Source: Front Oncol. 2025 Apr 17;15:1537611. doi: 10.3389/fonc.2025.1537611 (PMC12043674; doi:10.3389/fonc.2025.1537611)

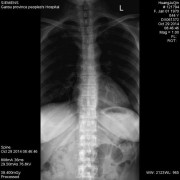

Supplement: Supplementary file 1 [file Image1.jpg]

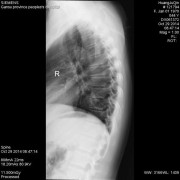

Supplement: Supplementary file 2 [file Image2.jpg]

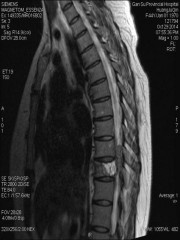

Supplement: Supplementary file 3 [file Image3.jpg]

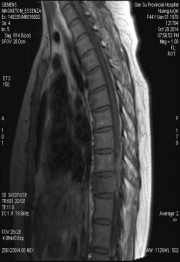

Supplement: Supplementary file 4 [file Image4.jpg]

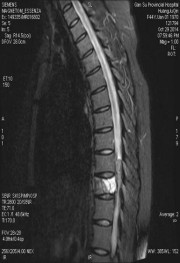

Supplement: Supplementary file 5 [file Image5.jpg]

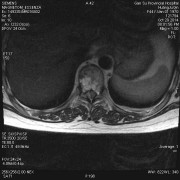

Supplement: Supplementary file 6 [file Image6.jpg]

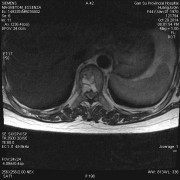

Supplement: Supplementary file 7 [file Image7.jpg]

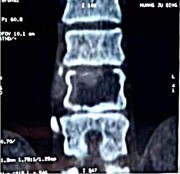

Supplement: Supplementary file 8 [file Image8.jpg]

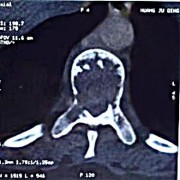

Supplement: Supplementary file 9 [file Image9.jpg]

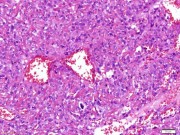

Supplement: Supplementary file 10 [file Image10.jpg]

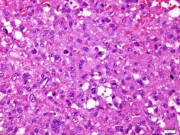

Supplement: Supplementary file 11 [file Image11.jpg]

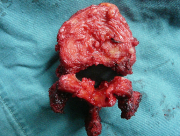

Supplement: Supplementary file 12 [file Image12.png]
